# A Single-center Retrospective Analysis of the Effect of Radium-223 (Xofigo) on Pancytopenia in Patients with Metastatic Castration-resistant Prostate Cancer

**DOI:** 10.7759/cureus.6806

**Published:** 2020-01-28

**Authors:** William P Skelton, Samantha W Dibenedetto, Shiyi S Pang, Kelsey Pan, Jacob L Barish, Adaeze Nwosu-Iheme, Long Dang

**Affiliations:** 1 Oncology, H. Lee Moffitt Cancer Center and Research Institute, Tampa, USA; 2 Internal Medicine, University of Florida, Gainesville, USA; 3 Oncology, University of Florida, Gainesville, USA; 4 Oncology, Ochsner Health System, Baton Rouge, USA

**Keywords:** radium-223, xofigo, prostate cancer, castration-resistant prostate cancer

## Abstract

Introduction

Radium-223 (Xofigo, Bayer Pharmaceuticals Inc., Whippany, NJ) has been shown to increase overall survival in patients with metastatic castration-resistant prostate cancer (mCRPC), via the phase 3 ALpharadin in SYMPtomatic Prostate CAncer (ASLYMPCA) study. Hematologic side effects of radium-223 included all-grade anemia in 31% of the patients, thrombocytopenia in 12%, and neutropenia in 5%, and persistent pancytopenia noted in 2%. However, the incidence seen in our institutional clinical practice is higher than that reported in the literature.

Methods

A retrospective analysis was performed by analyzing patients with mCRPC who received Xofigo at the University of Florida Health Shands Hospital (UF Health Shands) in a three-year span. Data collected included complete blood count (CBC), ECOG (Eastern Cooperative Oncology Group) functional status, kidney and liver function, evidence of bony disease on imaging, prior chemotherapy regimens, total radiation dose, and prostate-specific antigen (PSA).

Results

Twenty-three patients received Xofigo at UF Health, and one was lost to follow-up. Sixteen patients (73%) completed the full course (six doses) of Xofigo, while six did not. Ten patients (45%) developed pancytopenia, with two recovering counts within eight months while the other eight had persistent cytopenias (six of which were transfusion-dependent). Older age and higher ECOG score correlated with increased risk of pancytopenia. In addition, a higher percentage of patients who received prior radiation therapy were more likely to develop pancytopenia (90% vs 75%).

Conclusions

We found a higher rate of Xofigo-induced pancytopenia in our patient population than the 2% reported in the literature, albeit with a limited sample size, This may influence clinical decision making in the treatment of mCRPC, as pancytopenia may preclude patients from other survival-prolonging therapies. Factors such as age, functional status, and prior radiation therapy have to be considered prior to Xofigo treatment.

## Introduction

Prostate cancer is the second leading cause of cancer-related deaths in men in the United States [1]. Castration-resistant prostate cancer (CRPC) is defined by the progression of disease despite treatment with androgen deprivation therapy (ADT), 90% of which ultimately proceeds to have bone metastases [2]. Current treatment of metastatic CRPC (mCRPC) can include anti-androgens (abiraterone, enzalutamide), taxane-based chemotherapy, immunotherapy (sipuleucel-T, pembrolizumab), and radiation [3-6].

Radium-223, or Xofigo (Bayer Pharmaceuticals Inc., Whippany, NJ), was approved by the FDA for use in 2013 for CRPC in patients with symptomatic bone metastases in the absence of visceral involvement after the phase III ALpharadin in SYMPtomatic Prostate CAncer (ALSYMPCA) trial [7]. Radium-223 is a targeted alpha-particle-emitting agent that preferentially binds to the newly formed bone matrix, such as in metastatic bone lesions, causing cytotoxic dsDNA breaks to those targeted cells. Xofigo increased overall survival [median: 14.4 vs. 11.3 months; hazard ratio (HR): 0.70; 95% confidence interval (CI): 0.58-0.83; p: <0.001], along with time to first symptomatic skeletal event (15.6 months vs. 9.8 months; HR: 0.66; 95% (CI): 0.52-0.83; p: 0.0001) compared to placebo with bisphosphonates in patients with symptomatic bone metastases and no visceral involvement [8].

The most common side effect of Xofigo is cytopenia. Common non-hematological adverse events include nausea, vomiting, fatigue, constipation, peripheral edema, bone pain, and weight loss. In the ALSYMPCA trial, 31.2% of the total patients receiving Xofigo experienced some degree of anemia (compared to 30.6% of placebo), 11.5% had thrombocytopenia (compared to 5.6% of placebo), 5.0% had neutropenia (vs. 1% of placebo), and 4.2% had leukopenia (vs. 1% of placebo) based on the Common Terminology Criteria for Adverse Events (CTCAE), version 3.0 [7]. However, the incidence of grade-3-4 myelosuppression was low. Grade-3-4 thrombocytopenia occurred in 9% of patients taking Xofigo (compared to 3% taking placebo). Only one patient (<1%) out of 253 treated with Xofigo with no history of previous docetaxel treatment experienced grade-5 thrombocytopenia. Incidences of grade-3-4 anemia (12.8% in Xofigo vs. 13.0% in placebo) and neutropenia (1.8% in Xofigo vs. 0.7% in placebo) were comparable between the two groups [7]; 2% of patients treated with Xofigo experienced bone marrow failure or persistent pancytopenia throughout the duration of the follow-up period, compared with no patients treated with placebo. Among the patients taking Xofigo, 4% permanently discontinued therapy due to bone marrow suppression, vs. 2% in the placebo group [9].

At our institution, clinical practice has suggested a higher frequency of persistent pancytopenia in patients with mCRPC following Xofigo treatment than reported in the literature. This analysis serves to calculate the incidence of pancytopenia following treatment with Xofigo for mCRPC at our institution as well as to identify predictive factors and thereby help impact future therapy directions in this patient population. 

## Materials and methods

We conducted a retrospective review of patients with mCRPC who were treated with radium-223 (Xofigo) at the University of Florida Health Shands Hospital (UF Health Shands) in a three-year span (from January 2014 to January 2017) to determine the overall incidence of hematologic complications following treatment with Xofigo. This study was approved by the UF Institutional Review Board (IRB201703335), which included a waiver of individual patient consent. A retrospective analysis identified 27 patients with mCRPC who received Xofigo, of which four patients were treated at outside facilities and 23 were patients who received Xofigo at UF Health Shands. The patients who were not treated at UF Health Shands were excluded.

We performed a detailed review of the patients’ individual electronic medical records for their age, race, complete blood count (CBC: hemoglobin, platelet count, and white blood cell count), Eastern Cooperative Oncology Group (ECOG) score of functional status, kidney function (creatinine), liver function (alanine aminotransferase, aspartate aminotransferase, and total bilirubin), alkaline phosphatase level, evidence of bony disease on imaging scans, prior chemotherapy (and if so, which regimens), prior radiation therapy (and if so, total dose), and prostate-specific antigen (PSA) prior to the initiation of standard-dose Xofigo 55 kBq/kg intravenously. All-grade hematologic adverse events based on the CTCAE, version 3.0, were recorded following treatment with Xofigo to determine the incidence of pancytopenia (hemoglobin: <13.5 g/dL, platelet count: <150,000, and white blood cell count: <4.0 k/mm3) in our patient population. Descriptive data were presented as numbers and percentages. Data were analyzed using Breslow-Day and log-rank methods. A p-value of <0.05 was considered statistically significant. All statistical analysis was conducted using the Statistical Analysis System (SAS, Cary, NC) software (version 9.4). 

## Results

Overall, 23 patients met the inclusion criteria, with one patient lost to follow-up after receiving the first dose of Xofigo (and thus not included in baseline characteristics). Demographics for patients that did and did not develop pancytopenia following treatment with Xofigo are included in Table 1. Baseline characteristics were similar to that of the ALSYMPCA trial. There were no statistically significant differences in baseline characteristics, including age, ECOG Performance Status (PS), or prior chemotherapy and radiation between the group that developed pancytopenia compared to the group that did not develop pancytopenia. All patients had bony disease present prior to the initiation of Xofigo.

**Table 1:** Baseline patient characteristics ECOG PS: Eastern Cooperative Oncology Group Performance Status

Characteristic	Pancytopenic patients (n = 10)	Non-pancytopenic patients (n = 12)
Median age (range), years	74 (59–86)	69 (49–88)
Race, n (%)		
White	10 (100%)	9 (75%)
Black	0 (0%)	2 (17%)
Hispanic	0 (0%)	1 (8%)
ECOG PS, n (%)		
0	0 (0%)	1 (8%)
1	5 (50%)	5 (42%)
≥2	4 (40)	3 (25%)
Unknown	1 (10%)	1 (8%)
Prior chemotherapy, n (%)	10 (100%)	12 (100%)
Prior radiation therapy, n (%)	9 (90%)	9 (75%)

Baseline laboratory values, including hemoglobin, platelet count, white blood cell count, alkaline phosphatase, and PSA prior to treatment with Xofigo are included in Table 2. There were no statistically significant differences between the groups with respect to these values.

**Table 2:** Baseline laboratory values *Values in parentheses represent the range

Median biochemical values	Pancytopenia patients (n = 10)	Non-pancytopenia patients (n = 12)
Hemoglobin, g/dL	13.0 (8.3–14.6)*	12.5 (10.3–15.3)*
Platelet count, k/mm^3^	186 (123–270)*	199 (125–260)*
White blood cell count, k/mm^3^	5.5 (3.0–8.1)*	5.5 (4.2–8.5)*
Alkaline phosphatase, IU/L	92 (45–391)*	82 (47–521)*
Prostate-specific antigen, ng/mL	12.9 (0.06–1,000)*	11.0 (0.1–175)*

Sixteen patients (70%) completed therapy with six doses of Xofigo, and six patients (26%) discontinued therapy (mean/median = 3 doses). One patient (4%) was lost to follow-up after treatment initiation. The most common reason for discontinuation of Xofigo was disease progression with a transition to palliative care in three patients, followed by the development of pancytopenia in two patients, with the last patient discontinuing treatment due to gastrointestinal side effects. 

Out of the 23 patients who received Xofigo at UF Health Shands, 22 patients were analyzed, with the 23rd patient lost to follow-up. All patients had previously received treatment for prostate cancer, and 19 patients (83%) had received one or more of the other FDA-approved agents for mCRPC prior to Xofigo. These agents are docetaxel, abiraterone, enzalutamide, cabazitaxel, and sipuleucel-T, which along with Xofigo make up the list of agents that have shown survival benefit in mCRPC [3-6]; 82% of the patients had received prior radiation therapy, either external beam radiation to the prostate or skeletal radiation for symptomatic bone metastases.

Nine patients (41%) developed pancytopenia, and 12 patients (55%) did not develop pancytopenia. One patient (5%) had pancytopenia present prior to treatment initiation, which was persistent after the conclusion of treatment. All patients had at least one cytopenia present at the conclusion of treatment (100%). Prior to treatment initiation, eight patients (35%) had normal white blood cell, hemoglobin, and platelet counts (44% in the group that developed pancytopenia vs. 33% in the group that did not), 11 patients (48%) had one cytopenia (22% vs. 67%, respectively), and three patients (13%) had two cytopenias (33% vs. 0%, respectively).

Specifically, 14 patients (64%) developed anemia, of which nine were CTCAE grade 1-2 (41%), five were grade 3-4 (23%), and none was grade 5 (0%). Ten patients developed thrombocytopenia (46%), with seven having grade 1-2 (32%), three having grade 3-4 (14%), and none having grade 5 (0%). With regard to leukopenia, 11 patients (50%) developed the condition, with two patients having grade 1-2 (9%), nine patients having grade 3-4 (41%), and none having grade 5 (0%). For neutropenia specifically, three patients experienced it (14%), with two having grade 1-2 (9%), and one having grade 3-4 (5%) (Table 3). No patients experienced febrile neutropenia.

**Table 3:** Hematologic adverse events among study populations

		Skelton et al., 2019, %, (n= 22)	Hoskin et al., 2014, %, (n = 600)	Parker et al., 2018, %, (n = 600)
Hematologic adverse events				
Anemia	All	63.6	31.2	31.2
	Grades 1–2	40.9	18.4	18.0
	Grades 3–4	22.7	12.8	13.2
	Grade 5	0	0	0
Thrombocytopenia	All	45.5	11.5	11.5
	Grades 1–2	31.9	5.0	5.0
	Grades 3–4	13.6	6.3	6.5
	Grade 5	0	0.2	0
Neutropenia	All	13.6	5.0	5.0
	Grades 1–2	9.1	2.5	2.8
	Grades 3–4	4.5	2.5	2.2
	Grade 5	0	0	0
Leukopenia	All	50.0	4.2	N/A
	Grades 1–2	9.1	2.8	
	Grades 3–4	40.9	1.4	
	Grade 5	0	0	

Of the 10 patients who developed pancytopenia, two patients (20%) completely recovered counts within eight months of receiving their last dose of Xofigo. Eight patients (80%) had persistent cytopenias with a median follow-up of four months, of which six required subsequent transfusion of blood products with a total of 48 units of packed red blood cells (mean = 8) and 25 units of pooled platelets (mean = 4).

Of the 12 patients who did not develop pancytopenia, five patients (42%) developed anemia, two patients (17%) developed leukopenia, and one patient (8%) developed thrombocytopenia. Four patients (33%) had bi-cytopenia present; however, no patients developed more than one new cytopenia as a result of treatment. All patients had persistent mild anemia beyond the completion of Xofigo; however, none required a transfusion as a result of treatment with Xofigo. 

On average, patients that developed pancytopenia completed fewer cycles of Xofigo than those that did not develop pancytopenia (4.8 ± 1.5 cycles in the pancytopenia group vs. 5.5 ± 1.1 cycles in the non-pancytopenia group).

One patient died from complications of hemorrhagic brain metastases in the setting of moderate thrombocytopenia within three months of the last dose of Xofigo; no other patients died as a direct complication of pancytopenia or pancytopenia-related complications; there was no incidence of neutropenic fever either. Seven patients did not receive further courses of chemotherapy (docetaxel, abiraterone, enzalutamide, cabazitaxel, or sipuleucel-T) after treatment with radium-223. In two of these cases, the patient did not receive further chemotherapeutic agents due to death (one from treatment-related hematologic complications). One patient was unable to receive more chemotherapy due to poor performance status and thrombocytopenia. Another patient was enrolled in hospice due to pain-related issues. One patient preferred not to receive chemotherapy and opted for a PD-1 inhibitor. Finally, two patients did not receive any of the agents above further due to being in remission at last contact.

Age of ≥70 [odds ratio (OR): 2.40; p: 0.15] ECOG PS of ≥2 (OR: 1.60; p: 0.31), and prior radiation (OR: 3.0; p: 0.18) correlated with an increased risk of development of pancytopenia. Although not statistically significant, this suggests that older age, higher ECOG PS, and exposure to prior radiation treatment may increase the risk for subsequent development of pancytopenia. Additionally, any cytopenia present prior to the initiation of Xofigo did not suggest a significantly increased risk for developing pancytopenia (OR: 1.16; p: 0.43).

## Discussion

In this retrospective analysis of patients who received Xofigo for the treatment of mCRPC, we found significantly higher incidences of hematologic adverse events than previously reported in the literature. Of the 23 patients who received Xofigo, five patients (22%) experienced CTCAE grade-1 or higher pancytopenia. As per the clinical definition of pancytopenia (leukopenia, anemia, and thrombocytopenia), 10 patients (44%) developed pancytopenia. Of all the 23 patients, 10 (44%) had at least one CTCAE-defined grade-3/4 hematologic adverse event.

Parker et al. conducted a three-year safety analysis of the ALSYMPCA trial and found that grade-3/4 thrombocytopenia was more prevalent in the group that received Xofigo (7%) compared to the group that received placebo (2%). There were similar rates of grade-3/4 anemia (13% and 13%, respectively), and neutropenia (2% and 1%, respectively). With regard to all hematologic adverse events, thrombocytopenia was more prevalent in the Xofigo group (12%) compared to the placebo group (6%), and neutropenia was also more prevalent in the Xofigo group (5%) compared to placebo (1%). Anemia had the same prevalence in the Xofigo group and placebo (31% and 31%, respectively) [10].

Comparing our hematologic adverse events to the ALSYMPCA trial and the safety analysis by Parker et al. [7,11], there are several differences in incidence. We found a higher incidence of anemia, thrombocytopenia, and leukopenia compared to both studies. The largest discrepancies between our cohort and the other two large-scale studies were our grade-1-2 hematologic adverse events, specifically anemia (41% vs. 18%/18%, respectively) and thrombocytopenia (32% vs. 5%/5%, respectively). While our study had roughly two-fold higher incidences of grade-3-4 hematologic adverse events compared to the other two studies, adverse events among the studies are even more pronounced when examining the grade-1-2 discrepancies.

Baseline demographics of our patient population are similar to those reported in the literature; however, there are a few distinct differences that may have contributed to a higher incidence of hematologic adverse events in our patient population. Most notably, all of our patients had received prior chemotherapy with either docetaxel, abiraterone, enzalutamide, cabazitaxel, or sipuleucel-T prior to the administration of Xofigo, compared to the reported prior docetaxel use of 43% in ALSYMPCA trial. It was not reported if patients in this study had received other chemotherapeutic agents prior to Xofigo. Additionally, 83% of our patients had also received prior radiation therapy. As both chemotherapy and radiation can have myelosuppressive effects, prior exposure in our patient population may have contributed to more severe myelosuppression following the administration of Xofigo. Furthermore, our patient population had relatively higher ECOG PS (48% with ECOG ≥2) compared to patients in the ALSYMPCA trial (13% with ECOG ≥2) and similar studies, suggesting that worse baseline functional status may contribute to more hematologic adverse effects and delayed marrow recovery following treatment with Xofigo. With our cohort size being so small, it is difficult to extrapolate; however, there was at least one patient who was not able to receive further treatments following Xofigo. Although Xofigo has improved overall survival with a good safety profile and is being utilized earlier in patients with CRPC with bone metastases, the rare side effect of bone marrow failure should be considered in the clinical decision-making with the patient as it has the potential to affect future treatment options [11-12]. Although not statistically significant, factors such as age, functional status, and prior radiation therapy are important to keep under consideration prior to Xofigo treatment.

There are two main limitations to our study. First, due to the small number of patients who documented administration of Xofigo at our institution in the time frame covered, our study was unable to draw sweeping conclusions or perform detailed subgroup analysis. Nevertheless, we were able to use the cases of our patients to illustrate important points. Second, our study is a retrospective look at the patients who received Xofigo, and no comparison was done to similar patients who did not receive Xofigo. As the institution in question is a tertiary referral center, it is conceivable that cytopenias of all types are higher in this population regardless of Xofigo use.

Nevertheless, our study shows that Xofigo may have higher bone marrow toxicities in actual clinical practice than in the clinical trial setting, where patient selection is tightly controlled. It would be interesting to evaluate larger patient cohorts from both academic and community practices to determine how patients do with Xofigo upfront vs. later in the treatment sequence for mCRPC.

## Conclusions

The purpose of this study is not to define new and precise statistics in the incidence of hematologic adverse events with use of Xofigo, but to test a hypothesis noted during clinical practice: the incidence of hematologic adverse events appears to be higher than previously reported in the phase III ALSYMPCA trial (2-3%). Our data suggest that the incidence may be much higher, which may influence clinical decision-making in the treatment of CRPC. Indeed, in Bayer’s product monograph for Xofigo, in its “Warnings and Precautions” section, there are minimum hematologic counts recommended prior to initial and subsequent administrations of Xofigo. This is an important first step that acknowledges the potential serious hematologic adverse effects of Xofigo. Furthermore, one patient was contraindicated from receiving other survival-prolonging therapy after Xofigo treatment, at least partly due to thrombocytopenia, which may have been a consequence of Xofigo therapy. In summary, our results suggest that the possibility and consequences of bone marrow failure in Xofigo may be much more serious and common than previously reported, and should play a larger role in informed consent and shared decision-making. Further studies are necessary to elucidate this observation. 
